# Integrative Discovery Through Network Pharmacology and Molecular Docking Approaches of Phenolic Compounds Isolated from *Torreya nucifera* to Treat Rheumatoid Arthritis

**DOI:** 10.3390/ijms262311629

**Published:** 2025-11-30

**Authors:** Duc Dat Le, Thinhulinh Dang, Vinhquang Truong, Soojung Yu, Seung-Hwa Yang, Moon-Hee Choi, Mina Lee

**Affiliations:** 1 College of Pharmacy and Research Institute of Life and Pharmaceutical Sciences, Sunchon National University, 255 Jungangno, Suncheon 57922, Jeonnam, Republic of Korea; ddle@scnu.ac.kr (D.D.L.); 1220173@s.scnu.ac.kr (T.D.); 1243011@s.scnu.ac.kr (V.T.); 2 Department of Natural Cosmetics Science and Smart Beautytech Research Institute, Sunchon National University, 255 Jungangno, Suncheon 57922, Jeonnam, Republic of Korea; 1223002@s.scnu.ac.kr; 3 SUMSUMBIO Co., Ltd., Nano Bio Research Center, Jeonnam Bioindustry Foundation, 123 Nanosandan-ro, Jangseong 57248, Jeonnam, Republic of Korea; ysh8694@sumsumbio.com (S.-H.Y.); cmh2347@sumsumbio.com (M.-H.C.)

**Keywords:** *Torreya nucifera*, phenolics, rheumatoid arthritis, network pharmacology, NFκB pathway

## Abstract

*Torreya nucifera*, a consumable botanical species native to the southern regions of Korea, has a history of ethnopharmacological application to treat inflammatory conditions. This study employed feature-based molecular networking and integrated with the GNPS database to separate and identify ten phenolic compounds from *T. nucifera*. We further investigated the therapeutic potential of these isolated compounds and their structural features in the context of rheumatoid arthritis. Protein–protein interaction networks, constructed using compound–target and disease-associated target data, identified NFKB1, RELA, and TNFRSF1A as key hub genes. Gene Ontology (GO) enrichment analysis revealed inflammatory response as the most significantly modulated biological process. Kyoto Encyclopedia of Genes and Genomes (KEGG) pathway analysis indicated that the NF–kB signaling pathway is the most critical of the regulatory cascades influencing the pathogenesis of rheumatoid arthritis. Molecular docking studies verified strong binding affinities between the identified compounds and key target proteins. Furthermore, Western blot results validate that *T. nucifera* extract suppressed the LPS-activated NF–kB signaling pathways by inhibiting p65 and IκBα phosphorylation. The results underscore the significance of *T. nucifera* and its anti-inflammatory properties in relation to rheumatoid arthritis, establishing a scientific basis to formulate plant-based products within functional foods, nutraceuticals, and therapeutic interventions targeting rheumatoid arthritis.

## 1. Introduction

Rheumatoid arthritis (RA) is a chronic systemic autoimmune disorder that affects approximately (0.5 to 1) % of the global population, predominantly women, and acts as a major cause of disability and reduced quality of life [1]. The disease is characterized by persistent synovial inflammation and progressive joint destruction, which are driven by the infiltration of immune cells, such as T–cells, B–cells, macrophages, and neutrophils [2]. Pro-inflammatory cytokines, including tumor necrosis factor–α (TNF–α), interleukin (IL)–1β, and IL–6, play central roles in sustaining the inflammatory environment within the joints. These cytokines activate intracellular signaling cascades, notably NF-κB, JAK/STAT, and MAPKs, enhancing the production of inflammatory mediators, chemokines, and matrix metalloproteinases that perpetuate tissue injury [3]. Chronic inflammation in RA causes cartilage and bone erosion, while also contributing to systemic complications, such as cardiovascular disease, osteoporosis, and chronic fatigue [4]. Natural products and medicinal plants with demonstrated immunomodulatory properties are increasingly being investigated as alternative or complementary options to manage RA.

*Torreya nucifera* (Taxaceae), known as “비자(榧子)” in Korean traditional medicine, has long been used in East Asia to treat diseases. Extracts and constituents from *T. nucifera* modulated inflammation-related diseases. Several reports indicate the suppression of proinflammatory mediators (e.g., nitric oxide, prostaglandin E_2_) via the downregulation of iNOS and COX-2 [5], inhibition of NF-κB activation, and attenuation of AP-1 signaling (including p38 and JNK) pathways [6], which are central to cytokine production and inflammatory gene expression [7]. Previous study revealed that fraction of *T. nucifera* seeds showed potent anti-inflammatory effects toward cytokines and mediators through different AP-1 and NF-κB signaling pathways [8].

This study used various chromatographic and spectroscopic techniques to discover the molecular network-guided separation and identification of 12 chemical constituents from *T. nucifera* seeds. Furthermore, the study examined the potential mechanisms of action of the isolates against rheumatoid arthritis (RA), by integrating discovery of gene–gene, compound–gene, and gene–signaling pathway interactions for RA treatment through network pharmacology and molecular docking methodologies. Western blot assay confirmed the inhibitory effect of *T. nucifera* on NF-κB signaling pathways. The physicochemical properties of these compounds were also assessed by evaluating their physicochemical properties.

## 2. Results

### 2.1. Discovering Chemical Constituents via Molecular Network Discovery

**Figure 1:**
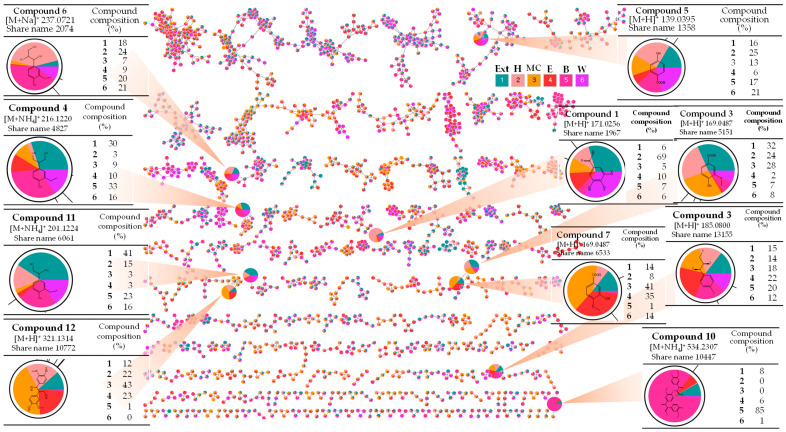
Compound annotations of phenolics identified from *T. nucifera* extract by application of GNPS-FBMN.

The application of Feature-Based Molecular Networking (FBMN), in conjunction with the Global Natural Products Social (GNPS) Molecular Networking platform, is significantly enhancing the process of annotating compounds, which is receiving significant focus in the realm of metabolomic analysis [9]. The GNPS–FBMN systematically arranges intricate mass spectrometry data into networks, with nodes symbolizing compounds and edges linking molecules that share structural similarities. GNPS–FBMN facilitates the rapid and precise annotation of compounds, even within complex mixtures, leveraging fragmentation patterns and spectral similarities. This approach enables the identification of compounds within mixtures, significantly enhancing the overall count of detected metabolites. This study encompassed the collection of high-resolution tandem mass spectrometry data regarding extracts and fractions via data-dependent acquisition experiments. The detailed mass spectra were employed for analysis through the interpretation of mass fragmentation (Table 1), in conjunction with the mass isotope patterns obtained from the GNPS platform (Figure 1).

Moreover, the chemical annotation was further interpreted by examining retention time, ultraviolet spectra, and their unique fragmentation patterns, compared to the respective standards.

### 2.2. Identification of Phenolic Compounds (***1***–***12***) from the Ethanolic Extract of T. nucifera

**Figure 2:**
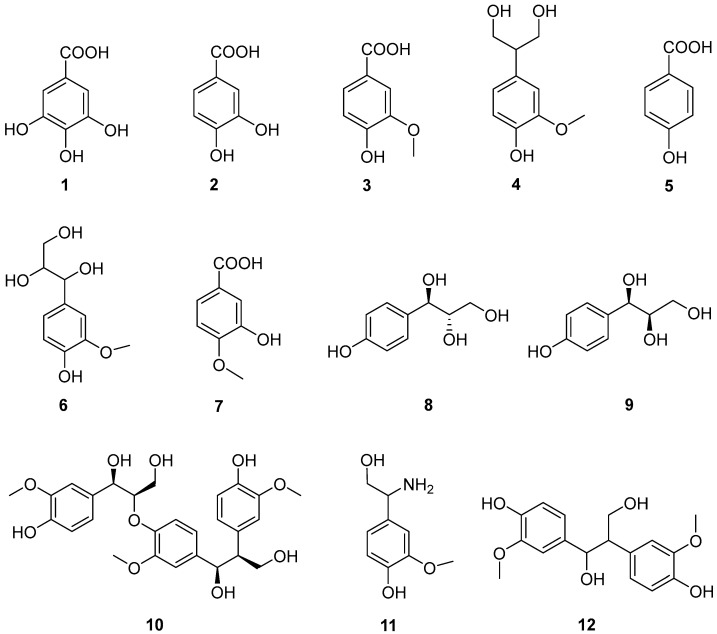
Chemical structures of compounds (**1**–**12**) isolated from *T. nucifera* seeds.

Phenolic compounds are widely recognized for their powerful antioxidant properties, which help protect cells from oxidative stress and reduce the risk of chronic diseases. These bioactive molecules, which are abundant in fruits, vegetables, and teas, exhibit notable anti-inflammatory, anti-aging, and antiproliferative effects that contribute to overall health and well-being [10]. Multiple studies have highlighted that the regular consumption of phenolic-rich foods supports cardiovascular health, and may lower the incidence of hypertension, diabetes, and neurodegenerative disorders. As a result, phenolic compounds are considered essential dietary components for disease prevention and the promotion of long-term human health [11]. Various chromatographic methods were employed to separate 12 phenolic compounds (Figure 2), guided by a molecular network approach. Their structures were elucidated using multiple spectroscopic techniques and spectrometry data and comparing the data with that reported from previous reports. As a result, these compounds were identified as gallic acid (**1**) [12], protocatechuic acid (**2**) [13], vanillic acid (**3**) [14], junipediol A (**4**) [15], 4–hydroxybenzoic acid (**5**) [16], (7*R*, 8*S*)–guaiacylglycerol (**6**) [17], isovanillic acid (**7**) [18], *threo*–1–(4–hydroxyphenyl)–glycerol (**8**) [19], *erythro*–1–(4–hydroxyphenyl)–glycerol (**9**) [19], leptolepisol D (**10**) [20], β–amino–3–methoxy–4–hydroxybenzen–ethanol (**11**) [21], and 1,2–bis(4–hydroxy–3–methoxyphenyl)–1,3–propanediol (**12**) [22].

### 2.3. Investigation of the Mechanisms of Actions of Isolated Compounds and Targets

**Figure 3:**
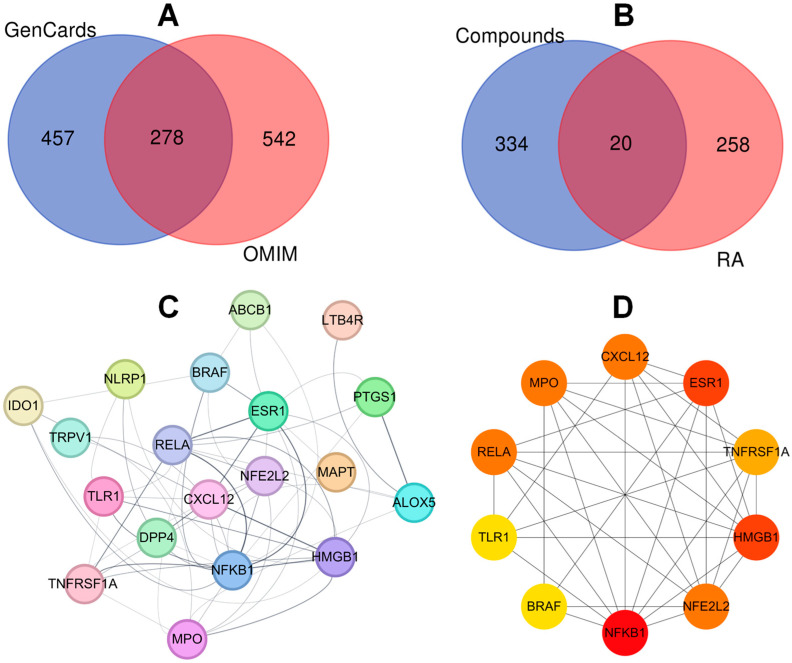
Protein–protein interaction network of intersecting target. Identification of overlapping targets against rheumatoid arthritis between GenCards and OMIM (**A**) and between compounds and RA (**B**). PPI network (**C**). Top rank target based on degree score (**D**).

To identify the therapeutic targets of the isolated compounds against rheumatoid arthritis (RA) (Figure 3), disease-associated targets were first collected from the GenCards and OMIM database. A total of 278 overlapping genes were obtained between the two databases, representing a robust set of RA-related targets (Figure 3A). Compound-associated targets of 12 isolated compounds from *T. nucifera* were also determined by employing the SEA database. Subsequently, disease and compound targets were analyzed, yielding 20 common targets out of 334 compound-related targets and 278 RA targets (Figure 3B). These overlapping genes were considered the most relevant mediators of the compounds’ potential against RA diseases. These targets were imported into the STRING web server to construct the protein–protein interaction (PPI) network consisting of 19 nodes (after removal of an unconnected node), and 56 edges of target actions (Figure 3C). Hub genes of NFKB1, HMGB1, ESR1, RELA, MPO, CXCL12, NFE2L2, and TNFRSF1A were identified, based on the degree score (Figure 3D).

### 2.4. KEGG Enrichment Analysis

**Figure 4:**
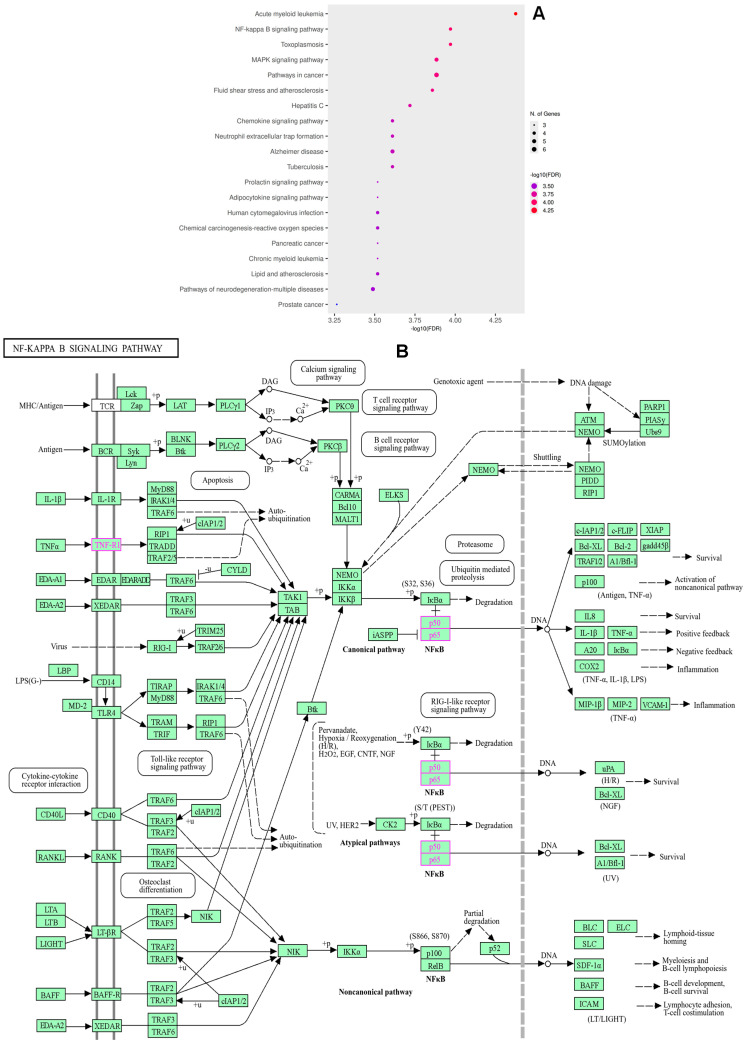
Top 20 KEGG pathways (**A**) and NF-κB signaling pathway (**B**).

KEGG pathway enrichment analysis revealed 142 significantly enriched signaling pathways associated with the overlapping targets of isolated compounds and RA-related diseases. Among the top 20 signaling pathways, the most enriched pathways included acute myeloid leukemia, NF-κB signaling pathway, toxoplasmosis, MAPK signaling pathway, and pathways in cancer (Figure 4). Other relevant pathways comprised the chemokine signaling pathway, neutrophil extracellular trap formation, adipocytokine signaling pathway, and lipid and atherosclerosis. Many of these pathways were highly significant, with multiple target genes mapped to each, underscoring their involvement in inflammation, immune regulation, and chronic disease progression. Among them, NF-κB signaling pathways showed highest enrichment scores targeting RA.

### 2.5. Gene Ontology (GO) Term Analysis

**Figure 5:**
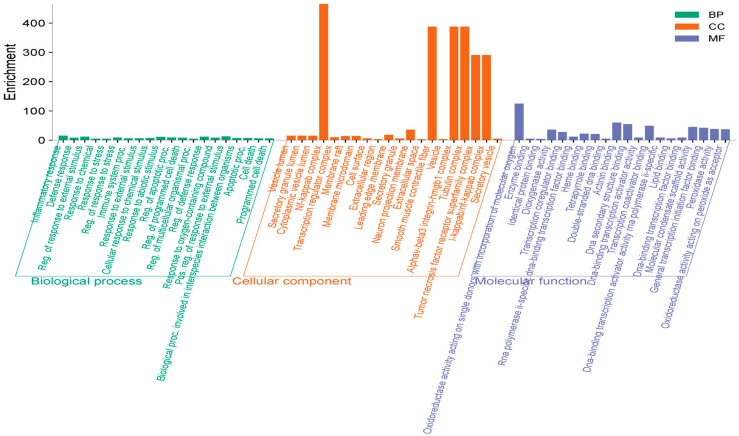
Top rank terms of gene ontology enrichment [Biological process (BP, green), cellular component (CC, orange), and molecular function (MF, blue)].

The overlapping targets were subjected to GO enrichment analysis to clarify their biological roles. The GO terms showed the presence of 1000 biological processes (BP), 92 cellular components, and 231 molecular functions, based on the intersecting targets. In the Biological Process (BP) category, the top 20 enriched terms were associated with inflammation and immune regulation, such as inflammatory response, defense response, regulation of response to external stimulus, immune system process, regulation of apoptotic process, and programmed cell death. These results indicate that the targets of the isolated compounds are closely involved in immune activation, inflammatory cascades, and the regulation of cell survival under pathological conditions. In the Cellular Component (CC) category, enriched terms were largely related to extracellular and membrane-associated structures. Highly significant terms included vesicle lumen, secretory granule lumen, cytoplasmic vesicle lumen, NF-κB complex, transcription regulator complex, cell surface, and extracellular space. These results suggest that the compound targets are distributed across compartments that are crucial for signal transduction, immune mediator secretion, and transcriptional regulation, particularly within NF-κB-related inflammatory signaling complexes. In the Molecular Function (MF) category, enriched terms were primarily associated with catalytic and binding activities (Figure 5). Significant terms included oxidoreductase activity, enzyme binding, dioxygenase activity, heme binding, transcription coactivator activity, lipid binding, and DNA-binding transcription factor activity. This indicates that the targets participate in redox regulation, transcriptional modulation, and enzyme–substrate interactions, highlighting their roles in oxidative stress defense and the regulation of gene expression in inflammatory pathways.

### 2.6. Construction and Analysis of Compound–Target–Pathway Interaction Network

**Figure 6:**
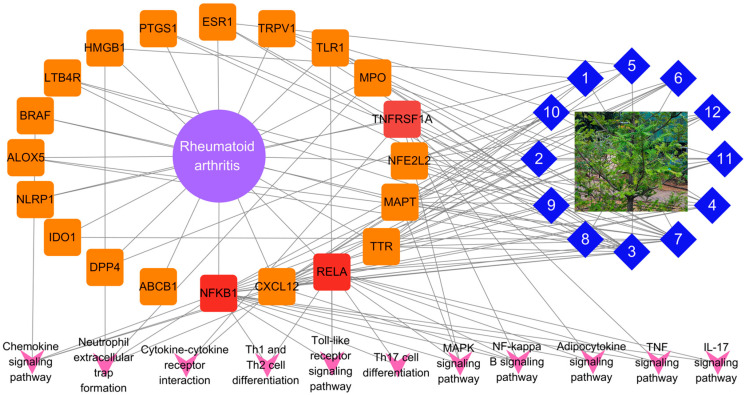
Network analysis of compounds in *T. nucifera* and their potential therapeutic targets in RA. The network illustrates the interactions between compounds (numbered blue diamonds) identified in *T. nucifera* extracts and protein targets associated with RA (purple circle). Orange-round rectangle represents target, with color intensity indicating key targets. Functional enrichment analysis reveals involvement in pathways indicated below the network.

A plant–compound–target–pathway interaction network constructed to visualize the multi-layered relationships among *T. nucifera*, its bioactive compounds, target genes, and the top 10 RA-related signaling pathways (Figure 6) shows that 12 compounds (blue diamonds) derived from *T. nucifera* are connected to multiple RA-associated targets (orange-reangle nodes). Among these targets, hub genes, such as NFKB1, RELA, TNFRSF1A, and CXCL12, exhibited strong connectivity, linking the compounds to several enriched pathways, such as NF-κB signaling, MAPK signaling, chemokine signaling, cytokine–cytokine receptor interaction, Toll-like receptor signaling, Th1/Th2 and Th17 differentiation, adipocytokine signaling, TNF signaling, and IL-17 signaling. Meanwhile, the integrated analysis of target genes and enriched KEGG pathways highlighted the complex molecular mechanisms underlying RA. Several hub genes, including TLR1, MPO, HMGB1, BRAF, MAPT, TNFRSF1A, CXCL12, RELA, and NFKB1, were identified as critical regulators of inflammation and immune dysregulation in RA. These targets collectively represent the key drivers of innate immune activation, oxidative stress, cytokine production, and immune cell recruitment, all of which contribute to synovial hyperplasia, cartilage degradation, and joint destruction [23,24].

**Figure 7:**
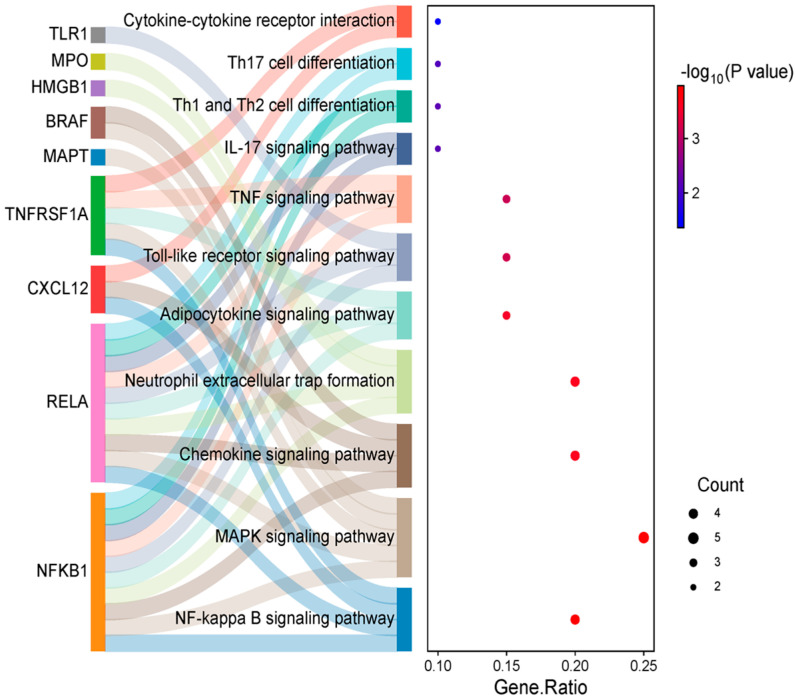
Interactions of targets and top rank KEGG pathways and target proteins by using Sankey plot.

A Sankey plot was created to clearly explain the targets and pathway interactions to treat RA. This plot revealed that NFKB1, RELA, and TNFRSF1A demonstrated multiple interactions with signaling pathways to treat RA (Figure 7). Therefore, these targets were selected for further docking studies.

### 2.7. Molecular Docking Verification

**Figure 8:**
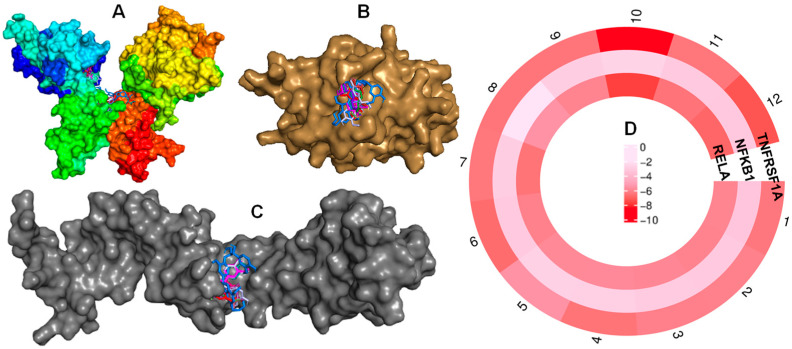
Compounds docked into the binding sites of Rela (**A**), NFKB1 (**B**), and TNFRSF1A (**C**) proteins. Circular heatmap of docked scores (**D**): color intensity represents strong binding affinity.

The potential effect toward RA-diseases of Compounds (**1**–**12**) were validated by molecular docking performance [25]. The results indicated that these compounds were bound to the receptors with negative binding energy values (Table S1, Supplementary Materials) through forming favorable interactions with the binding sites of receptors (Figure 8A–C). For TNFRSF1A and RELA proteins, compounds **1**–**12** showed high binding affinities based on their low docked scores, with scores below −5.63 kcal/mol, reflecting the strength of interaction between each compound and its receptors (Figure 8D). For NFKB1 protein, these compounds also display binding energies of about (−3.32 to −1.85) kcal/mol. Taken together, the docking results suggest that the *T. nucifera* components may interact with the key RA-related targets identified through network pharmacology. However, these findings are predictive in nature and require experimental validation to confirm their biological relevance.

### 2.8. T. nucifera Extract Reduced NFκB Activation by LPS-Stimulated RAW264.7 Cells

**Figure 9:**
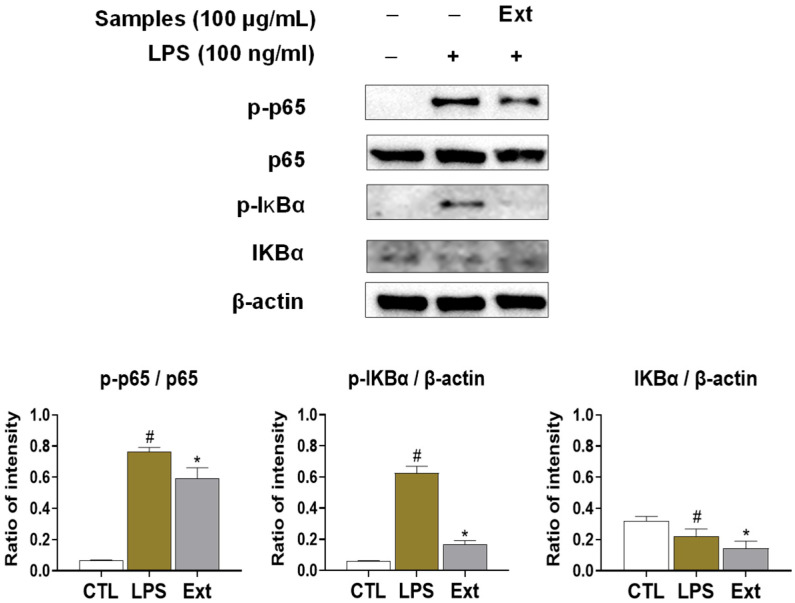
*T. nucifera* extract suppressed NF-κB signaling pathway in LPS-induced RAW 264.7 cells. Values are represented as means ± SEM (*n* = 3). # *p* < 0.05 vs. the control group; * *p* < 0.05 vs. LPS group in RAW 264.7 macrophages.

To confirm the effect of *T. nucifera* extract on the NFκB signaling pathway, we evaluated the potential effects of the extract on IκBα and p65 phosphorylation (Figure 9). Notably, LPS treatment remarkably increased the phosphorylation of both IκBα and p65 compared to the negative control (non-LPS treatment), indicating activation of the NFkB pathway. In contrast, pretreatment with Ext (100 µg/mL) significantly suppressed LPS-induced phosphorylation of p65 and IκBα. Moreover, the expression level of total IκBα, which was decreased after LPS stimulation, was restored by Ext treatment. These results suggest that Ext effectively inhibits the activation of the NF-κB pathway by preventing IκBα phosphorylation and degradation and subsequently reducing p65 activation, thereby contributing to its anti-inflammatory potential.

### 2.9. Physicochemical and Drug-likeness Assessment of the Isolated Compounds

Most of the compounds (**1**–**9**, **11**, and **12**) exhibited favorable physicochemical features and drug-likeness criteria (Table 2). Their molecular weights ranged (138 to 320) Da, with acceptable flexibility [number of rotational degrees (nRot) ≤ 6], balanced polarity [topological polar surface area (TPSA) value < 100 Å^2^, except for compound **10**], and moderate lipophilicity (−0.2 < SlogP < 1.1). These compounds also showed adequate hydrogen-bonding features [hydrogen bond acceptors (HBA): 2–6; hydrogen bond donors (HBD): 2–5] and a high quantitative estimate of drug-likeness (QED) scores ranging from 0.46 to 0.69. Notably, these compounds satisfied all the Lipinski, Pfizer, and GlaxoSmithKline (GSK) filters, indicating a low risk of attrition at the early stage. In contrast, compound 10 displayed deviations from standard drug-likeness criteria, including high molecular weight (516.5 Da), excessive polarity (TPSA = 158.3 Å^2^), high HBD (10) and HBA (6), and low QED score (0.21), resulting in a Lipinski violation. These properties suggest a lower oral bioavailability potential and reduced suitability as a lead compound, compared to the other members of the series. Therefore, except for compound **10**, these compounds may be considered promising drug-like candidates.

## 3. Discussion

The overlap of compound targets with RA-related genes highlights a multi-target mode of action, which is consistent with the complex pathophysiology of RA. The PPI network highlights the multi-target and multi-pathway therapeutic potential of *T. nucifera* against RA. Hub genes, such as NFKB1 and RELA, underscore the central role of NF-κB signaling in driving synovial inflammation, pro-inflammatory cytokine production, and immune cell activation in RA. By targeting these nodes, *T. nucifera* compounds may attenuate chronic inflammation and joint destruction. TNFRSF1A links the compounds to TNF–signaling, one of the most validated therapeutic pathways in RA, while CXCL12 indicates the modulation of chemokine signaling and immune cell recruitment into inflamed joints, which suggests that the isolated compounds may exert therapeutic effects primarily through the regulation of multiple inflammatory pathway signaling (via TNFRSF1A, RELA, and NFKB1) [26], leukotriene metabolism and immune cell activation (via ALOX5), and cytokine/chemokine signaling (via CXCL12 and PTGS1) [27]. Furthermore, ESR1 indicates the possible involvement of estrogen-related anti-inflammatory regulation, while NFE2L2 and HMGB1 imply additional mechanisms related to oxidative stress balance and innate immune modulation [28]. Thus, these findings suggest that the compounds isolated possess the ability to interfere with critical nodes in RA pathogenesis, acting synergistically to attenuate chronic inflammation, modulate immune cell activity, and protect against oxidative damage. These insights support the pharmacological relevance of the compounds, while also providing a mechanistic basis for their further evaluation as potential therapeutic agents against RA. Indeed, the GO enrichment terms highlight that the isolated compounds may exert anti-rheumatoid arthritis (RA) effects by modulating multiple biological processes, cellular localizations, and molecular functions. At the biological process level, the regulation of inflammation, immune response, and programmed cell death underscores the potential of the compounds to suppress chronic inflammation and prevent aberrant immune cell activity that drives RA pathogenesis. At the cellular component level, the enrichment of vesicle- and membrane-related terms indicates that these targets are positioned at critical sites of cytokine secretion and signaling complex formation, which are central to RA-associated joint inflammation [29]. Furthermore, the enrichment of the NF-κB complex aligns with the protein–protein interaction (PPI) analysis, supporting the potential role of the compounds in inhibiting NF-κB mediated inflammatory signaling. At the molecular function level, the enrichment of oxidoreductase and dioxygenase activities suggests that the compounds may also regulate oxidative stress, which is closely linked to RA progression through reactive oxygen species (ROS)-mediated joint damage [30]. In addition, the enrichment of transcription factor binding and coactivator activity indicates that these compounds may influence gene expression programs that are associated with immune and inflammatory regulation. Furthermore, the KEGG enrichment terms provide insights into the mechanistic basis by which the isolated compounds may exert therapeutic effects against RA. Enrichment of the NF-κB signaling pathway is particularly important, as NF-κB is a central regulator of inflammatory responses and synovial hyperplasia in RA.

Finally, the plant–compound–target–pathway interaction network demonstrates that *T. nucifera* acts through a systems-level mechanism that involves multiple compounds simultaneously regulating key immune and inflammatory pathways. This network pharmacology perspective provides strong evidence that the therapeutic effects of *T. nucifera* in RA are mediated not by a single compound or pathway, but rather through the synergistic regulation of interconnected molecular processes.

**Figure 10:**
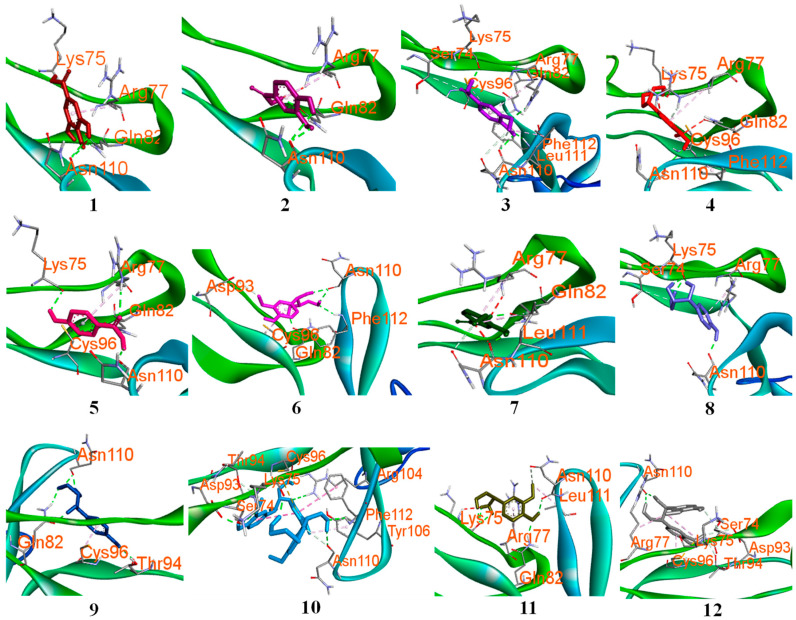
Three-dimensional interactions of compounds (**1**–**12**) with binding sites of TNFRSF1A protein (PDB ID: 1EXT).

Molecular docking analysis revealed that these isolated compounds displayed interactions with key residues at the binding pocket of protein by forming multiple hydrogen bonds. For protein (PDB ID: 1EXT), compounds showed interactions with key residues, such as SER74, LYS75, ARG77, ASN110, and LEU111, at the binding sites [31]. In particular, compound **10** showed the lowest docked score of −8.48 kcal/mol (Figure 10), by interacting with a large number of key residues, SER74, LYS75, ASP93, CYS96, and ASN110, through five hydrogen bonds.

Notably, compounds **1**, **4**, **8**, and **11** showed interactions with two key residues, LYS75 and ASN110, by forming hydrogen bonds. Compounds **2** and **5** demonstrated hydrogen bonds with three key residues of LYS75, ARG77, and ASN110 at the binding pocket of proteins. For 1VKX protein (Figure S1, Supplementary Materials), compounds **1**, **5**, **7**, **8**, **9**, and **10** showed interactions with key amino acids, such as LYS221, VAL244, ARG246, and GLN247, through forming hydrogen bonding at the binding sites in the interaction junction of NF–kB p50/p65 heterodimer [32], suggesting their potential effect to make them promising lead molecules for inhibiting NF–kB (p50/p65) heterodimer complex to check the cytokine storms in humans. Therefore, molecular docking indicates that the compound can bind RELA (p65) and TNFRSF1A with relatively favorable scores (both < −5.90), consistent with the potential to interfere with NF-κB and TNF–α signaling pathways and the activity or localization of the p65 subunit of NF-κB mechanisms that are highly relevant to rheumatoid arthritis pathogenesis [33]. It is proposed that *T. nucifera* may prevent p65 nuclear translocation, impairing p65–p50 dimer formation or blocking upstream TNFR1 activation. These in silico results are hypothesis-generating and require experimental validation. Our experimental results verified that *T. nucifera* extract effectively suppressed LPS-induced activation of the NF-κB signaling pathway, a central regulator of inflammatory responses. The observed reduction in p65 phosphorylation and restoration of IκBα expression indicate that Ext interferes with the canonical NF-κB activation cascade by stabilizing IκBα and preventing its degradation. Since the phosphorylation and subsequent degradation of IκBα are essential steps leading to the nuclear translocation of p65 and transcription of pro-inflammatory genes, these findings suggest that Ext may inhibit the production of inflammatory mediators through blockade of this signaling axis. Similar inhibitory patterns on NF-κB activation have been reported for several bioactive plant-derived compounds, supporting the role of natural extracts as modulators of inflammation. Therefore, the suppression of NF-κB activation by *T. nucifera* extract provides a mechanistic basis for its anti-inflammatory potential and highlights its promise as a natural therapeutic candidate for inflammatory disorders.

Previous studies have shown that many isolated compounds provide considerable therapeutic potential against rheumatoid arthritis (RA) by regulating critical inflammatory signaling pathways. Specifically, compound **1** was observed to enhance RA pathophysiology by modulating immune responses through the downregulation of pro-inflammatory cytokines [34]. Compound **2** was demonstrated to reduce the progression of rheumatoid arthritis (RA) by suppressing the Akt/mTOR and NF-κB signaling pathways [35]. In contrast, compound **3** exhibited anti-arthritic effects by decreasing inflammatory responses through the simultaneous inhibition of the NF-κB and MAPK pathways [36]. Furthermore, compound **5** reduced RA-related inflammation by inhibiting the NF-κB/caspase–1 cascade [37]. Moreover, other compounds (**6** and **10**) have been reported to inhibit the synthesis of inflammatory mediators, hence reinforcing their extensive anti-inflammatory characteristics [38,39]. Therefore, isolated compounds may regulate multi-target modulators of immunological and inflammatory signals, facilitating the reduction in RA pathogenesis.

The physicochemical landscape of the isolated compounds shows that compounds **1**–**9**, **11**, and **12** are privileged for orally active molecules, indicating drug-likeness. Consistent compliance with Lipinski-, Pfizer-, and GSK-based constraints moderates lipophilicity. Their polarities suggest crossing biological membranes to reach disease-relevant intracellular targets. Their favorable QED distribution suggests that these scaffolds are compatible with early-phase drug development rules, and in a chemical space enriched for successful therapeutics.

## 4. Materials and Methods

### 4.1. Plant Materials

The seeds of *T. nucifera* were collected and identified by Professor Mina Lee (College of Pharmacy, Sunchon National University). Afterwards, a voucher specimen (SCNUP 28) was prepared and stored at the Pharmacognosy Laboratory (College of Pharmacy, Sunchon National University) in Korea.

### 4.2. Extraction and Preparation of T. nucifera Extract and Fractions

Dried seeds of *T. nucifera* (10 kg) were ground into a powder and extracted via sonication with 40% EtOH at 25 °C for 120 min. The resulting extract was concentrated in vacuo to yield a crude extract (390 g). This crude extract was then sequentially partitioned with n-hexane (H), methylene chloride (MC), ethyl acetate (E), and n-butanol (B), producing five distinct fractions: H (136 g), MC (35.1 g), E (86.5 g), B (53 g), and an aqueous residue (W, 75 g).

### 4.3. Analytical Conditions and Construction of Feature-Based Molecular Network

Extract and fractions of *T. nucifera* were dissolved in the high-grade methanol followed by sonification for 20 min at 50 °C. Then, these solutions were filtered and stored for further analysis. The analytical procedures were carried out by using instruments reported in our previous publication [40] without modification. The raw mass data of extract and fractions were then converted into the mzmine formats by using the Mzmine 3.9.0 program. The output files were successfully uploaded to the Global Natural Product Social Molecular Networking (GNPS) web site to create a feature-based molecular networking file, which were further visualized by using Cytoscape software (ver. 10.1) [40,41]. Compound annotation was conducted by accessing the GNPS structural database and in-house database. The workflow can be found in the following GNPS (https://gnps.ucsd.edu, accessed on 15 September 2025) repository with a task ID_4e43dd4e047c4e32a334d70c6f9d3ddd.

### 4.4. Separation of Compounds

The E fraction (86.5 g) was subjected to silica gel column chromatography using a gradient solvent system of dichloromethane and methanol (5:1 to 0:100, *v*/*v*) to yield nine subfractions (E1–E9). Subfraction E6 was further purified using preparative reversed-phase medium-pressure liquid chromatography (RP-MPLC) on a YMC Triart ODS C_18_ column (250 × 10 mm, 5 µm). A gradient of methanol in water (containing 0.1% formic acid) from 5% to 100% was used over 60 min. UV detection was performed at 210, 224, and 254 nm, which resulted in eight subfractions (E6A–E6H). Compounds **8** (1.1 mg) and **12** (0.8 mg) were subsequently isolated from subfractions E6D and E6H, respectively, using preparative thin-layer chromatography (TLC) on silica gel 60 RP-18 F_254S_ plates with a mobile phase of methanol/distilled water (1:2.5, *v*/*v*).

The B fraction (53 g) was initially separated into five subfractions (B1–B5) using a Dianion HP-20 column with a gradient elution of methanol in water (0–100%). From subfraction B3, compounds were isolated via preparative high-performance liquid chromatography (prep-HPLC) on a YMC C_18_ column (10 × 250 mm, 5 µm). The mobile phase was a gradient of water containing 0.1% formic acid (A) and acetonitrile (B) from 5% to 100% B over 40 min at a flow rate of 3.0 mL/min. This separation yielded compounds **1** (*t*_R_ 18.9 min, 0.8 mg), **2** (*t*_R_ 23.4 min, 0.4 mg), and **5** (*t*_R_ 21.6 min, 1.3 mg). Separately, subfraction B5 was fractionated on a Sephadex LH-20 column (2.5 × 33 cm) with an H_2_O-CH_3_OH gradient (75:25 to 0:100, *v*/*v*), yielding compound **4** (*t*_R_ 21.0 min, 0.5 mg) and four subfractions (B5C–B5F). Compounds **3** (*t*_R_ 36.0 min, 0.7 mg), **6** (*t*_R_ 16.6 min, 0.9 mg), **9** (*t*_R_ 12 min, 0.4 mg), and **10** (*t*_R_ 29 min, 1.0 mg), were then isolated from subfraction B5D by prep-HPLC using YMC C_18_ column (10 × 250 mm, 5 μm) from 5% to 100% for 40 min. Similarly, compounds **7** (*t*_R_ 16.0 min, 0.3 mg) and **11** (*t*_R_ 10 min, 0.6 mg) were isolated from subfraction B5C under these same prep-HPLC conditions above.

### 4.5. Network Pharmacology

#### 4.5.1. Definition of Compound and Disease Targets

The structures of compounds (**1**–**12**) were entered into the Pubchem database to get the smiles before loading them into the Similarity Ensemble Approach web tools (https://sea.bkslab.org/, accessed on 15 September 2025) to retrieve the targets of compounds [42]. Rheumatoid arthritis related targets were acquired from the Gene Cards (https://www.genecards.org/, accessed on 15 September 2025) and Online Mendelian Inheritance in Man (OMIM) (https://omim.org/, accessed on 15 September 2025). The intersecting targets between compound and disease targets were obtained through Venny 2.1.0 (https://bioinfogp.cnb.csic.es/tools/venny/, accessed on 15 September 2025) online platform.

#### 4.5.2. Construction of PPI Network

Key targets involved in treating rheumatoid arthritis were inputted into the STRING database (https://string-db.org/, accessed on 15 September 2025) by setting species as homo sapiens and confidence score upper 0.7. The output data obtained from the STRING was then sent to the Cytoscape (Ver. 10.1) using STRING plugin for visualization and interconnection of targets interactions network. The core targets were identified by using the Cytohubba plugin Cytoscape software based on degree score.

#### 4.5.3. GO and KEGG Enrichment Terms

Functional enrichment analysis was conducted based on Kyoto Encyclopaedia of Genes and Genomes (KEGG) and Gene Ontology (GO). KEGG pathway enrichment and GO enrichment terms included biological process, molecular function, and cellular component, were caried out in SHINYGO0.82 (http://bioinformatics.sdstate.edu/go/, accessed on 15 September 2025) database by set cut-off values *p* < 0.05 and FDR < 0.01. Enrichment visualization was also done in SRPLOT. The KEGG mapper (https://www.genome.jp/kegg/mapper/, accessed on 15 September 2025) highlighted the top pathway and demonstrated its distinct molecular mechanism within pathway.

### 4.6. Molecular Docking In Silico Studies

To validate the binding mechanism between compounds and each core target protein, we performed molecular docking studies [43] to predict the binding affinity of those interactions. The structures of TNFRSF1A (PDB ID: 1EXT), RELA (PDB ID: 1VKX), an NFKB1 (PDB ID: 8TQD) proteins were downloaded from the RCSB Protein Data Bank (https://www.rcsb.org; accessed 25 October 2025). The names of compounds (**1**–**12**) were inputted into the Pubchem to get the 3D conformers. Protein and ligand preparation was performed by using the MGL tools. Docking score function of each ligand and protein was calculated by using the Autodock 4.2.6 algorithm. The scoring function incorporated van der Waals interactions, electrostatics, hydrogen bonding, desolvation effects, and torsional entropy contributions, facilitating precise predictions of ligand-binding modes and affinities. The grid-box dimension of protein was defined based on binding sites such as SER74, LYS75, ARG77, ASN110, and LEU110 [31] for 1EXXT protein, and druggable pockets of protein [32] for 1VKX protein. The root mean square deviation values between the original (JMR: 1-(2-bromo-4-chlorophenyl)-N-{(3S)-1-[(E)-iminomethyl]pyrrolidin-3-yl}methanesulfonamide) and redocked native ligands was 1.8099 Å (PDB ID: 8TQD), demonstrating the reliability and accuracy of the method employed. Binding affinities and interactions of ligand and binding sites were visualized using Pymol software (version 3.1.4.1) and Discovery Studio Visualizer 2021.

### 4.7. Western Blot Assays

To conduct the Western blot assays, we utilized Raw264.7 cell lines, which were obtained from the Korean Cell Lines Bank in Seoul, Korea, to evaluate the potential effect of *T. nucifera* extract. After 2 h pre-treatment, the cells were stimulated with lipopolysaccharide (LPS) at a concentration of 100 ng/mL and lysed with the addition of PRO-PREPTM from Intro Biotechnology in Seoul, Republic of Korea (Thermo Fisher Scientific’s Pierce™ Bradford Protein Assay Kit (n.d.)). The protein concentration was measured using the Pierce™ Bradford Protein Assay Kit. After transferring 20 μg of whole proteins to a PVDF membrane, they underwent SDS-PAGE separation using 8% and 10% acrylamide gel. Cell Signaling Technology of Danvers, MA, USA, supplied the antibodies p65 (#8242), p-p65 (#3033), and p-IκBα (#9246). Santa Cruz Biotechnology, Inc., Santa Cruz, CA, USA, supplied the IκBα (SC-371) antibodies. β-actin reference number (AB_2289199). The PVDF membranes were firstly incubated with antibody after being blocked with 5% skim milk for 2 h, followed protein transfer. The primary antibodies were diluted in 2.5% skim milk with a ratio of 1:1000 and left to incubate at 4 °C for 18 h. After that, the blots were washed three times with Tween20/Tris-buffered saline (T/TBS). Then, they were incubated with a secondary antibody that was conjugated with HRP and diluted 1:2000 in 5% skim milk for 2 h at room temperature. Finally, the blots were washed three times with T/TBS. As a means of detecting the expression of the target protein, the researchers turned to the Super Signal™ West Femto Maximum Sensitivity Substrate (Thermo Fisher Scientific, Waltham, MA, USA) [43].

### 4.8. Physiochemical, Pharmacokinetics, and Drug-likeness Profiles of Compounds

The smiles of compounds were inputted into the ADMESAR3.0 [44] to predict the physicochemical, medicinal, and pharmacokinetic properties by using web platform (https://lmmd.ecust.edu.cn/admetsar3/index.php, accessed on 15 September 2025).

### 4.9. Statistical Analysis

Data was represented as mean ±  standard error of the mean (*n* = 3). We used GraphPad Prism (GraphPad Software, version 8.0.2, Inc., San Diego, CA, USA) to conduct a one-way analysis of variance (ANOVA) and Dunnett’s post hoc test to examine values that were deemed statistically significant. When compared to the control groups, there were significant differences at ^#^ *p* < 0.05 and * *p* < 0.05

## 5. Conclusions

We isolated 12 phenolic compounds from the ethanolic extract of *T. nucifera* through a molecular network approach, then applied various spectroscopic techniques to elucidate the structures of the isolated compounds. Using the network pharmacology method, we investigated how the biological mechanisms of these isolated chemicals in *T. nucifera* regulate various targets and signaling pathways, in particular, the NF-κB signaling pathway, further verified by Western blot data in the treatment of rheumatoid arthritis. The results of molecular docking validation indicated that compounds **1**–**12** exhibited significant interactions with crucial hub genes associated with rheumatoid arthritis. Our findings present a detailed examination of the proposed mechanisms of the action of *T. nucifera* in the treatment of rheumatoid arthritis. Further studies focused on in vivo and clinical validation should be performed to validate the potential therapeutic benefits of these compounds and extracts of *T. nucifera*.

## Figures and Tables

**Table 1:** Tentatively identified compounds in the ethanolic extract of *T. nucifera* obtained by LC-MS/MS analysis.

No.	Compound	*t*_R_ (min)	Mass (*m/z*)	Adduct Ions	Molecular Formula	Fragment Ions (*m/z*)
1	Gallic acid *	2.42	171.0256	[M + H]^+^	C_7_H_6_O_5_	126.0544/109.0280
2	Protocatechuic acid *	5.01	155.0330	[M + H]^+^	C_7_H_6_O_4_	110.0597/93.0695
3	Vanillic acid *	6.90	169.0488	[M + H]^+^	C_8_H_8_O_4_	124.0388/93.0695
4	Junipediol A *	6.62	216.1220	[M + NH_4_]^+^	C_10_H_14_O_4_	134.0124/93.3847
5	4-Hydroxybenzoic acid *	2.05	139.0019	[M + H]^+^	C_7_H_6_O_3_	93.0444
6	(7*R*, 8*S*)-guaiacylglycerol *	2.82	237.0720	[M + Na]^+^	C_11_H_11_O_5_	123.0548/91.0540
7	Isovanillic acid *	8.79	169.0487	[M + H]^+^	C_8_H_8_O_4_	137.0591/93.0695
8	Threo-1-(4-hydroxyphenyl)-glycerol *	23.94	185.0800	[M + H]^+^	C_9_H_12_O_4_	124.0864
9	Erythro-1-(4-hydroxyphenyl)-glycerol	23.94	185.0800	[M + H]^+^	C_9_H_12_O_4_	124.0864
10	Leptolepisol D *	17.16	534.2311	[M + NH_4_]^+^	C_27_H_32_O_10_	214.1427
11	β-amino-3-methoxy-4-hydroxybenzen-ethanol	8.14	201.1225	[M + NH_4_]^+^	C_9_H_13_NO_3_	167.0332/135.1163
12	1,2-bis(4-hydroxy-3-methoxyphenyl)-1,3-propanediol *	19.31	321.1321	[M + H]^+^	C_17_H_20_O_6_	290.2677/259.2047/167.1060

* Mass data was identical to those of standards.

**Table 2:** Physicochemical and drug-likeness properties of isolated compounds.

Compound	Molecular Weight	nRot	HBA	HBD	TPSA	Drug-likeness	Lipinski	Pfizer	GSK
**1**	170.12	1	4	4	97.99	0.46	Pass	Pass	Pass
**2**	154.121	1	3	3	77.76	0.52	Pass	Pass	Pass
**3**	168.148	2	3	2	66.76	0.69	Pass	Pass	Pass
**4**	198.218	4	4	3	69.92	0.66	Pass	Pass	Pass
**5**	138.122	1	2	2	57.53	0.61	Pass	Pass	Pass
**6**	214.217	4	5	4	90.15	0.56	Pass	Pass	Pass
**7**	168.148	2	3	2	66.76	0.69	Pass	Pass	Pass
**8**	184.191	3	4	4	80.92	0.52	Pass	Pass	Pass
**9**	184.191	3	4	4	80.92	0.52	Pass	Pass	Pass
**10**	516.543	12	10	6	158.3	0.21	No	Pass	Pass
**11**	183.207	3	4	3	75.71	0.63	Pass	Pass	Pass
**12**	320.341	6	6	4	99.38	0.65	Pass	Pass	Pass

## Data Availability

The original contributions presented in the study are included in the article/Supplementary Materials, further inquiries can be directed to the corresponding author.

## Supplementary Materials

The following supporting information can be downloaded at: https://www.mdpi.com/article/10.3390/ijms262311629/s1.
