# Supplementary material for: Integrative Discovery Through Network Pharmacology and Molecular Docking Approaches of Phenolic Compounds Isolated from Torreya nucifera to Treat Rheumatoid Arthritis
Source: Int J Mol Sci. 2025 Nov 30;26(23):11629. doi: 10.3390/ijms262311629 (PMC12691866; doi:10.3390/ijms262311629)
Supplement: Supplementary file 1 [file ijms-26-11629-s001.zip › ijms-3998294-supplementary.pdf]

Supplementary materials

Integrative Discovery Through Network Pharmacology and Molecular Docking Approaches of Phenolic Compounds Isolated from Torreya nucifera to Treat Rheumatoid Arthritis

Duc Dat Le 1,2, Thinhulinh Dang 1, Vinhquang Truong 1, Soojung Yu 2, Seung-Hwa Yang 3, Moon-Hee Choi 3 and Mina Lee 1,2,*

1 College of Pharmacy and Research Institute of Life and Pharmaceutical Sciences, Sunchon National
University, 255 Jungangno, Suncheon, Jeonnam 57922, Republic of Korea; ddle@scnu.ac.kr (D.D.L.); 1220173@s.scnu.ac.kr (T.D.); 1243011@s.scnu.ac.kr (V.T.)

2 Department of Natural Cosmetics Science and Smart Beautytech Research Institute, Sunchon National
University, 255 Jungangno, Suncheon, Jeonnam 57922, Republic of Korea; 1223002@s.scnu.ac.kr

3 SUMSUMBIO Co. Ltd., Nano Bio Research Center, Jeonnam Bioindustry Foundation, 123 Nanosandan-ro, Jangseong, Jeonnam 57248, Republic of Korea; ysh8694@sumsumbio.com (S.-H.Y.);
cmh2347@sumsumbio.com (M.-H.C.)

* Correspondence: minalee@sunchon.ac.kr or minalee@scnu.ac.kr; Tel.: +82-61-750-3764; Fax: +82-61-750-3708

Table S1. Docked scores of compounds with target proteins

Compound \ Protein	TNFRSF1A	NFKB1	RELA
**1**	−6.45	−3.32	−6.05
**2**	−6.17	−3.13	−6.12
**3**	−6.22	−3.08	−5.99
**4**	−6.56	−2.87	−5.91
**5**	−5.63	−2.8	−5.93
**6**	−6.76	−3.15	−6.17
**7**	−6.48	−2.94	−6.58
**8**	−6.52	−1.85	−5.65
**9**	−6.53	−2.94	−6.08
**10**	−8.48	−2.63	−7.78
**11**	−6.51	−3.16	−6.57
**12**	−7.34	−3.2	−6.91

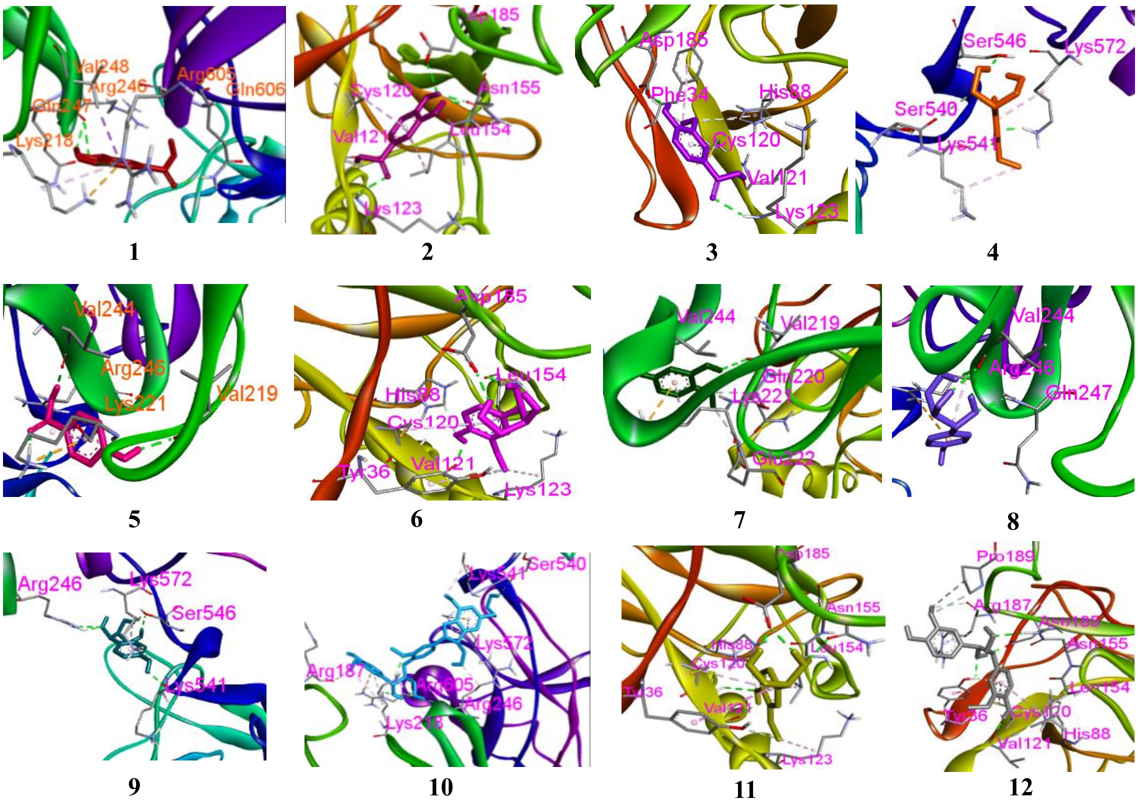

Figure S1. 3D interactions of compounds with amino acid when they were docked into RELA (P65) protein (PDB ID: 1VKX).

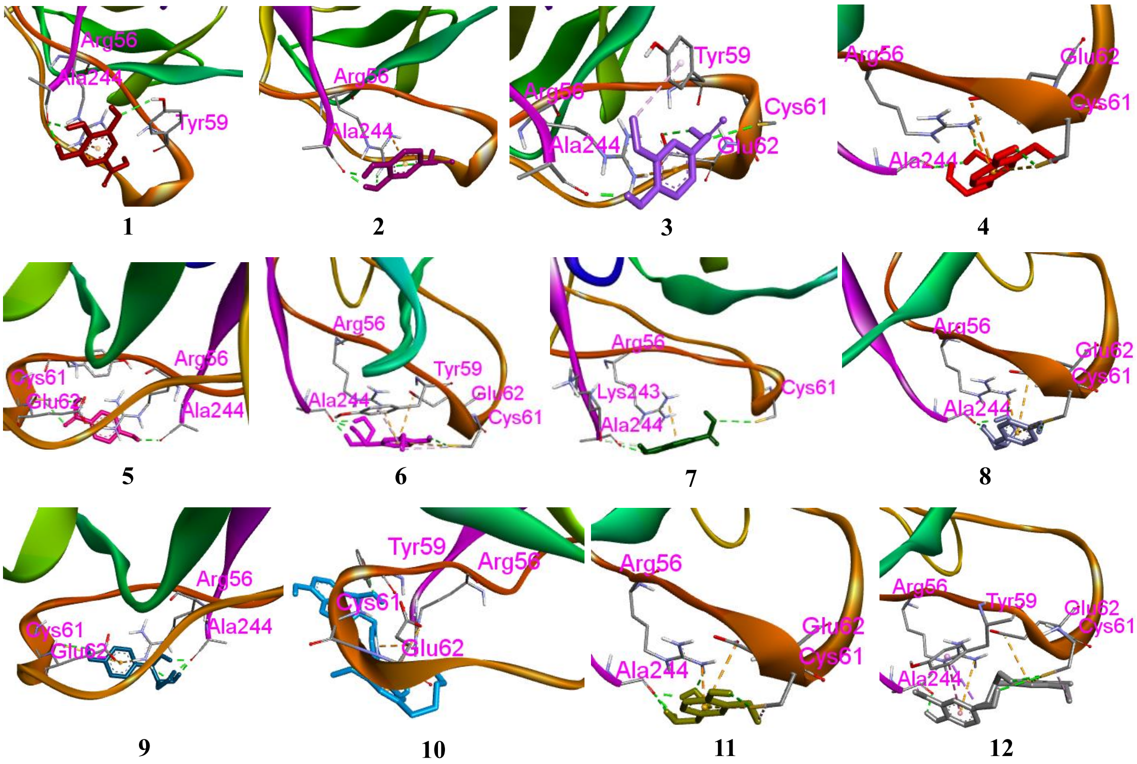

Figure S2. 3D interactions of compounds with amino acid when they were docked into NFKB1 protein (PDB ID: 8TQD).

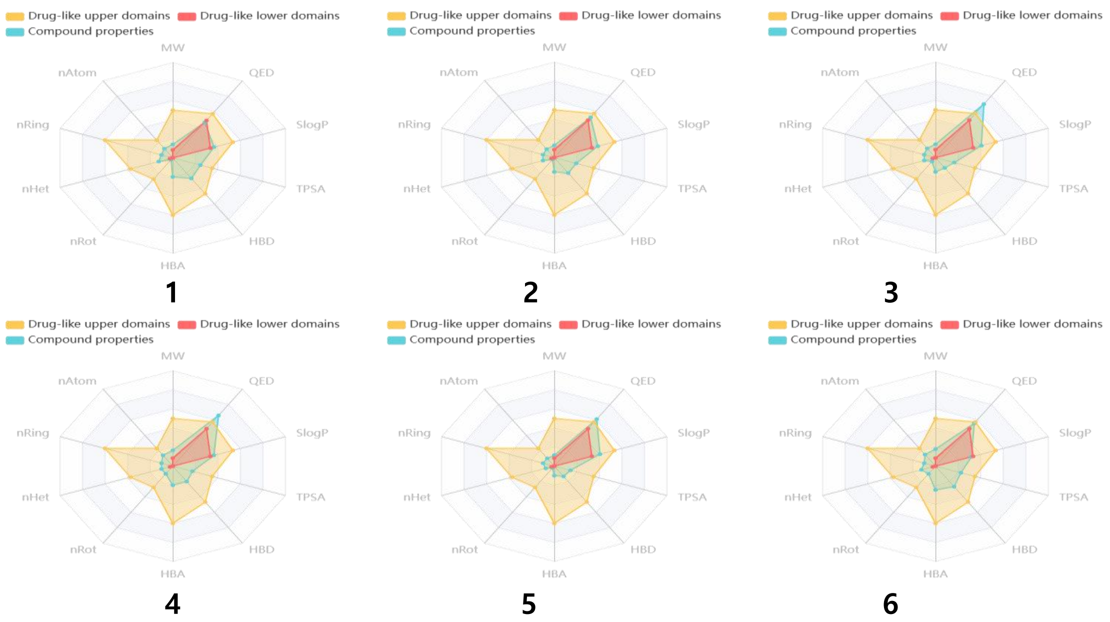

Figure S3. Radar plot illustrates the physicochemical profiles of the investigated compounds (**1**–**6**) in comparison with drug-likeness domains. The yellow shaded area represents the upper bounds of drug-like space, whereas the red shaded area denotes the lower bounds. The blue overlay indicates the measured properties of the compound. Axes correspond to molecular weight (MW), quantitative estimate of drug-likeness (QED), partition coefficient (SlogP), topological polar surface area (TPSA), hydrogen bond donors (HBD), hydrogen bond acceptors (HBA), number of rotatable bonds (nRot), number of heteroatoms (nHet), number of aromatic rings (nRing), and total atom count (nAtom). This comparative visualization enables an assessment of the compound's fit within the established drug-like chemical space.

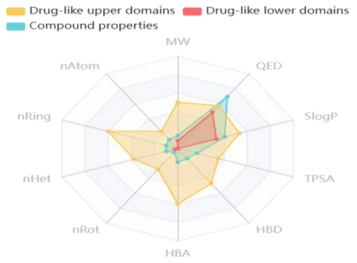

7

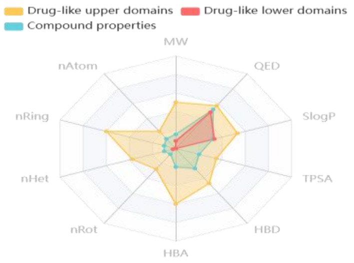

8

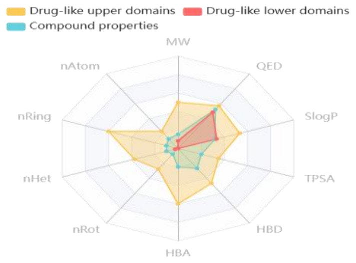

9

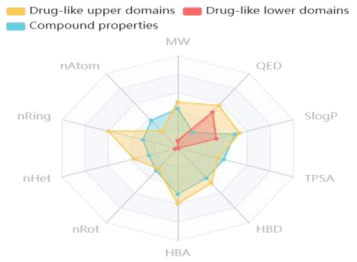

10

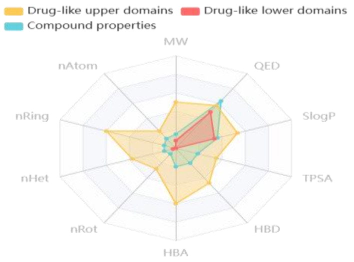

11

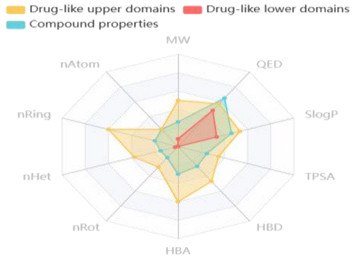

12

Figure S4. Radar plot illustrates the physicochemical profiles of the investigated compounds (**7–12**) in comparison with drug-likeness domains. The yellow shaded area represents the upper bounds of drug-like space, whereas the red shaded area denotes the lower bounds. The blue overlay indicates the measured properties of the compound. Axes correspond to molecular weight (MW), quantitative estimate of drug-likeness (QED), partition coefficient (SlogP), topological polar surface area (TPSA), hydrogen bond donors (HBD), hydrogen bond acceptors (HBA), number of rotatable bonds (nRot), number of heteroatoms (nHet), number of aromatic rings (nRing), and total atom count (nAtom). This comparative visualization enables an assessment of the compound's fit within the established drug-like chemical space.
